# Next generation antibiotic combinations to combat pan-drug resistant *Klebsiella pneumoniae*

**DOI:** 10.1038/s41598-024-53130-z

**Published:** 2024-02-07

**Authors:** Jan Naseer Kaur, Navaldeep Singh, Nicholas M. Smith, Jack F. Klem, Raymond Cha, Yinzhi Lang, Liang Chen, Barry Kreiswirth, Patricia N. Holden, Jürgen B. Bulitta, Brian T. Tsuji

**Affiliations:** 1 Center for Infectious Diseases Next Generation Therapeutics, University at Buffalo, Buffalo, NY USA; 2 Division of Clinical and Translational Therapeutics, School of Pharmacy and Pharmaceutical Sciences, University at Buffalo, Buffalo, NY USA; 3 Center for Discovery and Innovation, Hackensack Meridian Health, Edison, NJ USA; 4 Department of Pharmacotherapy and Translational Research, College of Pharmacy, University of Florida, Orlando, FL USA

**Keywords:** Antimicrobials, Microbiology

## Abstract

Antimicrobial resistance has emerged as one of the leading public health threats of the twenty-first century. Gram-negative pathogens have been a major contributor to the declining efficacy of antibiotics through both acquired resistance and tolerance. In this study, a pan-drug resistant (PDR), NDM-1 and CTX-M-15 co-producing isolate of *K. pneumoniae,* CDC Nevada, (*Kp* Nevada) was exposed to the clinical combination of aztreonam + ceftazidime/avibactam (ATM/CAZ/AVI) to overcome metallo-β-lactamases. Unexpectedly, the β-lactam combination resulted in long filamentous cell formation induced by PBP3 inhibition over 168 h in the hollow fiber infection model experiments with eventual reversion of the total population upon drug removal. However, the addition of imipenem to the two drug β-lactam combination was highly synergistic with suppression of all drug resistant subpopulations over 5 days. Scanning electron microscopy and fluorescence microscopy for all imipenem combinations in time kill studies suggested a role for imipenem in suppression of long filamentous persisters, via the formation of metabolically active spheroplasts. To complement the imaging studies, salient transcriptomic changes were quantified using RT-PCR and novel cassette assay evaluated β-lactam permeability. This showed significant upregulation of both spheroplast protein Y (SPY), a periplasmic chaperone protein that has been shown to be related to spheroplast formation, and penicillin binding proteins (PBP1, PBP2, PBP3) for all combinations involving imipenem. However, with aztreonam alone, *pbp1, pbp3* and *spy* remained unchanged while *pbp2* levels were downregulated by > 25%. Imipenem displayed 207-fold higher permeability as compared with aztreonam (mean permeability coefficient of 17,200 nm/s). Although the clinical combination of aztreonam/avibactam and ceftazidime has been proposed as an important treatment of MBL Gram-negatives, we report the first occurrence of long filamentous persister formation. To our knowledge, this is the first study that defines novel β-lactam combinations involving imipenem via maximal suppression of filamentous persisters to combat PDR CDC Nevada *K. pneumoniae*.

## Introduction

With the ever-climbing trajectory of multidrug resistance, infections by Gram-negative pathogens have become an incredible public health challenge^1,2^. Infections caused by *Klebsiella pneumoniae*, from the family of *Enterobacterales,* have been particularly problematic. *Klebsiella pneumoniae* (*Kp*) is an opportunistic Gram-negative pathogen that is capable of causing life threatening respiratory tract, vascular, nosocomial, and urinary tract infections in both immunocompromised and immunocompetent hosts^3,4^. Recently, studies have shown that up to 80% of *Kp* isolates can be resistant to first-line antibiotics^5,6^. This dramatically limits the therapeutic options. The emergence of carbapenemase producing strains in highly resistant backgrounds has left clinicians with a dearth of efficient biocidal regimens of antibiotics that are mechanistically driven.

Due to the remarkably high antibiotic resistance levels displayed by multi-drug resistant strains of *Kp*, infections caused by such strains are responsible for increased mortality rates ranging from 22% to as high as 77%^7,8^. Scientists worldwide are constantly striving to find novel drugs, new drug combinations, and innovative ‘cocktails’ to combat the *Kp* strains that already display a multi-drug resistance^9,10^. This situation is amplified even further in situations of *Kp* strains which are resistant to nearly all or all antibiotics. A clear example of these Gram-negative ‘superbugs’ are those that produce plasmid-mediated New Delhi Metallo-β-lactamase-1 (NDM-1) which is a cause for significant concern and urgency in the scientific community. This enzyme confers high-level resistance to carbapenems, and most other β-lactams (except aztreonam). No NDM-active β-lactamase inhibitor are currently available for clinical use. Thus, in the current study, we devised novel therapeutic strategies to treat a pan-drug resistant strain of *Kp-*CDC Nevada. This strain of *Kp* harbors 12 resistance genes (*bla*_NDM-1_, *bla*_CMY-6_, *bla*_CTX-M-15_ and *bla*_SHV-28_, *fosA, mphA, aacA4, rmtC, oqxA, oqxB, sul1, mphA*, insertional inactivation of *mgrB, gyrA and parC* mutations), and mutations in ramR; resistant to all available monotherapies. In addition to these heritable resistance mechanisms, development of persister phenotypes also attribute to recalcitrant infections and treatment failure. The morphotype changes during and following the antibiotic treatment are also related to persister formation which further confounds the therapeutic capabilities of drugs. Tolerance exhibited by persisters followed by regrowth after the drug removal has evolved as a leading cause of antibiotic treatment failure^11^. Taken together, in the face of pan-drug resistance, exploring strategies to target and eliminate persisters is of significant importance to combat the public health problem of Gram-negative ‘Superbugs.’ As one of the strategies, in this manuscript we ascertain the efficacy of co-administration of ceftazidime/avibactam and aztreonam with and without imipenem against the pan resistant *Kp-*CDC Nevada using hollow-fiber infection model (HFIM) studies.

## Methods

### Media, bacteria and antimicrobial agents

For all experiments, fresh Muller–Hinton Broth (MHB) (Difco, Detroit, MI, USA) was used. Total bacterial counts were determined by plating samples on Muller-Hinton agar (MHA) (Difco, Detroit, MI, USA) followed by incubation at 37 °C for 24 h. This study utilized an unusual pan resistant *Klebsiella pneumoniae*, that was isolated from a hip abscess of a U.S. patient (BioProject PRJNA391323, GenBank Accession number: CP022125.1-CP022128.1)^12^. Previous characterization of this isolate has shown that it harbors four known beta lactamase genes, including plasmid-mediated bla_NDM-1_ and bla_CMY-6_, as well as chromosomal bla_CTX-M-15_ and bla_SHV-2_. This strain was found to be non-susceptible to a range of drugs tested, including all beta-lactams, colistin, and tigecycline. For time- kill assays, fresh drug stocks of aztreonam and imipenem (AKScientific, Union City, CA, USA) were used at clinically relevant concentrations.

### Time-kill experiments

Time-kill studies were conducted as previously described^13^. Briefly, antibiotics were examined as monotherapy and in combination against our isolate. For starter cultures, isolates were sub-cultured onto MHA plate and incubated at 37 °C for 24 h. Cells were scraped and resuspended in MHB to a final concentration of 10^7^ CFU/ml in 20 ml final volume. Antibiotic solutions were added to achieve the desired drug concentrations (aztreonam, 64 mg/L; imipenem, 7.5 mg/L), whereupon tubes were incubated at 37 °C in a shaking water bath (180 rpm) for 24 h. Serial samples (0.2 ml) were collected aseptically at 0, 1, 2, 4, 6, and 24 h, with viable counting conducted immediately via serial dilution (using 0.9% saline) and spiral plating (WASP2 spiral plater; Don Whitley Scientific, Ltd., UK) of 50 μl of undiluted or appropriately diluted suspension onto MHA, followed by incubation at 37 °C.

### Hollow fiber infection model

*Kp* Nevada was investigated in a 10-day hollow fiber infection model (HFIM) using cellulosic cartridges (Cartridge C3008; FiberCell Systems Inc., Frederick, MD), as described previously^14^. The system was set up to simulate in vitro a 2 h half-life with a total system volume of distribution of 135 mL. For the regimens tested, drugs were administered up to 168 h, after which the remaining drug in the system was allowed to be washed out with a 2 h half-life. Bacterial samples were collected at 0, 1, 2, 4, 6, 24, 26, 28, 30, 48, 50, 52, 54, 72, 120, 168, 192, 198, 216 h for viable cell counting. Total bacterial counts and population analysis profiles (PAPs) were determined by sub-culturing 50 μL of serially diluted bacterial suspensions onto MHA alone for total bacterial counts or MHA containing polymyxin B (at 0.5, 2, 8, and 32 mg/L), aztreonam/avibactam (at 2/4 mg/L, 8/4 mg/L, and 32/4 mg/L), or ceftazidime (at 2 mg/L, 8 mg/L, and 32 mg/L) for PAPs. Aztreonam/avibactam PAPs were utilized over aztreonam/ceftazidime/avibactam based on the accepted mechanism of action of the combination, which is reliant on the predominant effects of aztreonam and avibactam.

### Fluorescence and scanning electron microscopy

Samples for imaging were collected from HFIM cartridges and time-kill reaction conicals prior to antibiotic exposure (0 h) and from a series of time points after treatment to observe changes in morphology over time. Cells were fixed with filtered glutaraldehyde (Electron Microscopy Sciences, Hatfield, PA, USA) to a final concentration of 2.4%, gently mixed, and stored at 4 °C for at least overnight before processing. For fluorescence, 5ul of the glutaraldehyde fixed sample was mixed with 1ul of SYTO 9 green, fluorescent nucleic acid stain and visualized on Leica DM 6B upright fluorescence microscope at 63×/1.4 oil immersion objective. The images were processed using Leica Application Suite X (LasX). For SEM, preserved cells were gently collected onto 0.2 um Nucleopore polycarbonate filters (Whatman, Sigma-Aldrich, St. Louis, MO, USA) by syringe-filtering using Swin-lok cartridges (Whatman, 25 mm). Cells were washed with 1× PBS and then dehydrated in a graded ethanol series, as follows: 8 ml 30% v/v, 50% v/v, 70% v/v, 80% v/v, 90% v/v, 95% v/v, 97.5% v/v, ethanol /deionized water for 10–15 min for each step. In the final exchange, 100% ethanol was passed through twice, incubating for 10 min each time. Samples were then dried with filtered hexamethyldisilazane (HMDS, 1–2 mL, 5 min). The Nucleopore filters were allowed to completely air-dry and the prepped samples were gold sputter-coated and imaged under a Carl Zeiss Auriga Focused Ion beam-Scanning Electron Microscope at 10,000x magnification.

### RNA isolation, cDNA synthesis and qRT-PCR

20 ml culture of *Kp* Nevada was incubated with and without pertinent drug concentrations for 2 h at 37 °C with shaking at 225 rpm. 15 ml aliquots were then removed, centrifuged to pellet, and resuspended in 1 ml of RNA STAT-60 (Tel-Test, Inc). About 0.2 ml of chloroform: iso-amyl alcohol 24:1 (Calbiochem) was added to each aliquot, microfuge tubes were inverted several times to mix, and centrifuged at 18,000× *g* for 30 min at 4 °C. The aqueous layer was removed and transferred to a new microfuge tube, to which 0.5 ml of isopropanol was added (Fischer Scientific), mixed by inversion and incubated overnight at − 20 °C to precipitate RNA. The next day, samples were centrifuged at 18,000× *g* for 30 min at 4 °C, supernatants were discarded, and the nucleic acid pellet was washed three times with 1 ml of 75% ethanol and centrifuged at 18,000× *g* each wash for 10 min. After the final wash, the supernatant was carefully removed and the pellet was air-dried, then dissolved in 30 µL of molecular biology grade water (Corning). Nucleic acid concentration was measured by Nanodrop. DNA was removed via the Invitrogen DNA-Free Kit, according to manufacturer’s protocol. DNAse-treated RNA was frozen at − 80 °C until use. RNA samples were DNase treated and reverse transcribed using the iScript cDNA synthesis kit (Bio-Rad) according to the manufacturer's instructions. The quantitative reverse transcription (qRT)-PCRs were performed in triplicate with the Applied Biosystems qPCRBIO SyGreen Blue Mix kit utilizing custom DNA oligonucleotide primers for each of the transcripts analyzed. The qRT-PCR reactions were performed in a final volume of 10 µl and cDNA was amplified using a CFX Connect Real-Time System (Bio-Rad). Analysis of qRT-PCR results was done using BioRad iQ5 software. Changes in RNA levels were quantitated using the comparative threshold cycle (CT) method. For analysis, proC and gyrA were chosen as the reference gene for normalization between samples as described previously^15^. For specificity, cDNA synthesis reactions minus reverse transcriptase were run in parallel as control. Additionally, no template controls were run to check for contamination. Average fold gene expression was plotted on the graph for each transcript with the standard error of mean in the error bars. All results shown reflect three biological replicates, each with three technical replicates of the qRT-PCR reaction. Oligonucleotide sequences for the transcripts verified are provided in Table S1.

### Permeability

Using a previously established LC–MS/MS protocol^16^, we quantified the permeability of aztreonam, ceftazidime, and imipenem (along with meropenem cefepime, and piperacillin as additional comparators) by measuring the degradation of each drug under permeability-limited (i.e., intact cells washed of extracellular beta-lactamase) and Michaelis–Menten conditions (i.e., lysed bacteria previously washed of extracellular beta-lactamase) when exposed to *Kp* Nevada^16–18^. Permeability coefficients of meropenem (AK Scientific, Union City, CA), imipenem (AK Scientific, Union City, CA), aztreonam (AK Scientific, Union City, CA), cefepime (AK Scientific, Union City, CA), ceftazidime (Sigma Aldrich, St. Louis, MO), and piperacillin (AK Scientific, Union City, CA) were quantified in *K. pneumoniae* isolates over 120 min. All drugs were tested at 1, 3, 10, and 30 mg/L with quality control concentrations of 0.8 and 8 mg/L to verify inter- and intra-day accuracy and precision of the assay. Protein precipitation was performed with methanol (including isotopically labeled internal standard at 3 mg/L). For the standard curve, 90 uL of PBS and 10 uL of the drug were added to final concentrations of 80, 8, 30, 3, 0.3, 10, and 1 mg/L. (See Supplemental Section) Data were analyzed using ordinary differential equations, with key parameters estimated for β-lactam specific permeability, intrinsic clearance (Cl_int_, defined as V_max_/K_m_), the drug “affinity” (K_m_). Modeling was performed using S-ADAPT/S-ADAPT_TRAN^19^.

## Results and discussion

The increasing risk for antibiotic treatment failure and relapsing infections by Gram-negative pathogens is largely a consequence of antimicrobial resistance and persistence^11,20,21^. Previous studies have elucidated that both the stationary phase persisters (type I) and continuously generated persisters (type II) are amongst the constituents of wild type population^22^. Irrespective of the instigating factors and mechanism of formation, bacterial persisters favor the survival of a subpopulation when exposed to antibiotics, hostile environments, or other stressors. A small subpopulation of persister cells can be detected even under favorable growth conditions, which can be extended after exposure to high concentrations of bactericidal agents^23,24^. Persisters have the ability to evade killing and exhibit high tolerance to drugs that require rapid growth for their bactericidal action by virtue of their decreased metabolic activity^25,26^. In this study, we observed robust spheroplast formation in pan-drug resistant (PDR) CDC Nevada *K. pneumoniae* in response to imipenem exposure. Additionally, we investigated alteration in expression of key genes responsible for morphology homeostasis in response to the drug exposure, which is an important aspect to identify and optimize targeted treatment/ prevention.

**Figure 1:**
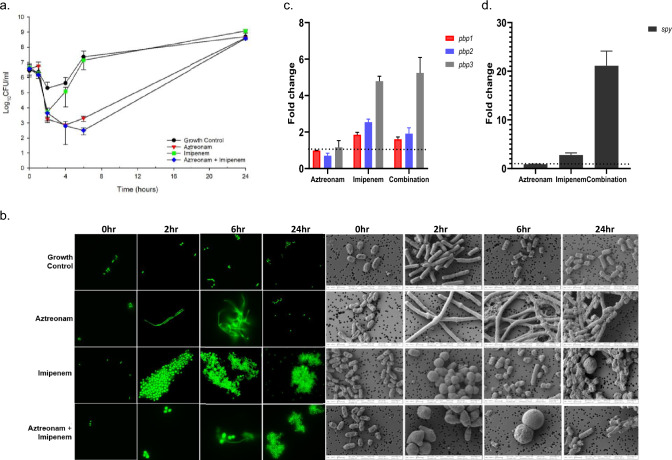
(**a**) Time-kill experiments of aztreonam (62 mg/L), imipenem (7.5 mg/L) and their combination against 10^7^ CFU/mL of XDR *Kp* Nevada. Experiments were performed in duplicate and standard deviations are indicated by error bars. (**b**) Confocal microscopy images of representative *Kp* Nevada at 0 h, 2 h, 6 h, and 24 h (A) no drug exposure, (B) exposure to aztreonam, (C) exposure to imipenem, (D) exposure to aztreonam plus imipenem. Glutaraldehyde fixed bacterial samples were stained using SYTO9 and imaged on Leica DM 6B upright fluorescence microscope using 63×/1.4 oil immersion objective. Scanning electron microscopy images of representative *Kp* Nevada cells at a magnification of 10,000x. Images were obtained from HFIM samples taken at indicated time and drug treatment as mentioned above. Glutaraldehyde fixed samples were dehydrated on 0.2 um nucleopore filters through graded ethanol washing and final hexamethyldisilazane (HMDS) addition. Samples were imaged using a Carl Zeiss Auriga Focused Ion Beam-Scanning Electron Microscope. (**c**), (**d**) At 2 h post exposure total RNA was extracted, reverse transcribed and analyzed by qRT-PCR for the expression of the indicated genes (n = 3). *proC* and *gyrA* expression was used for normalization. Fold gene expression in treatment arms was measured in comparison to the growth control.

PDR *Kp* Nevada grown on Mueller Hinton II Agar (MHA, Becton, Dickinson and Company; Franklin Lakes, New Jersey) was utilized for all experiments^27^. The MICs for *Kp* Nevada were confirmed for all antimicrobials used in this experiment via broth microdilution in duplicate according to CLSI guidelines (MIC_aztreonam_ > 16 mg/L, MIC_ceftazidime/avibactam_ > 32/4 mg/L, MIC_imipenem_ 32 mg/L)^28^. Time-kill studies were conducted as previously described^13^. Briefly, a starting inoculum of 10^7^ CFU/mL was targeted in all experiments based on observed bacterial concentrations in nosocomial pneumonia to uncover the antimicrobial regimens with the greatest activity as defined by undetectable bacterial counts at 24 h. Fluorescence and scanning electron microscopy were then used to explore the broader morphological changes in response to each of the key β-lactams driving bacterial killing. For preliminary investigation, *Kp* Nevada was exposed to 3 different drug treatments for 24 h that included imipenem (7.5 mg/L), aztreonam (62 mg/L), or the imipenem + aztreonam combination. Given the ability of *Kp* Nevada to produce carbapenemase, imipenem monotherapy was rendered ineffective (Fig. 1a). For aztreonam monotherapy and the aztreonam + imipenem combination, there was an initial 3 log_10_ reduction in total counts as compared to growth control up until 6 h (Fig. 1a). However, by 24 h, all the treatment arms had counts comparable to that of the no drug condition (Fig. 1a).

**Figure 2:**
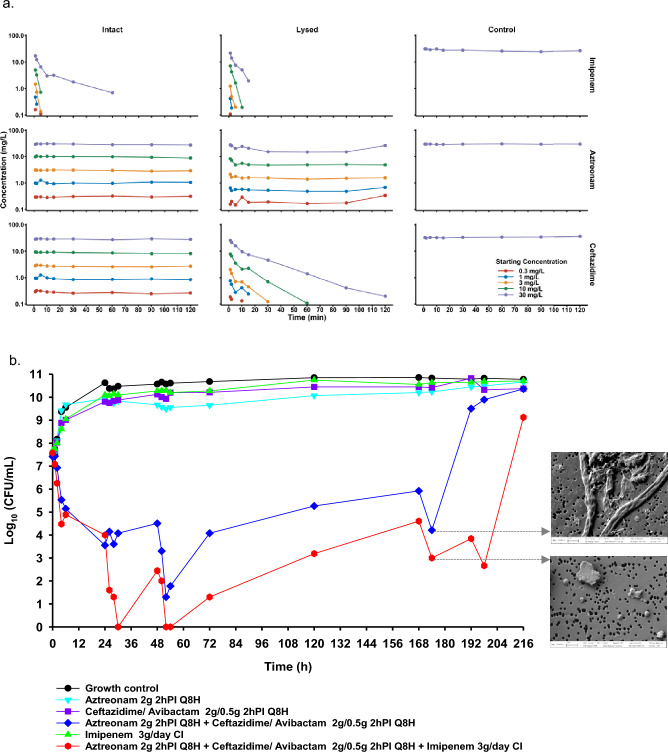
(**a**) Assessment of outer membrane permeability for imipenem, aztreonam, and ceftazidime under permeability-limited (‘Intact cell’ column), Michaelis–Menten (‘Lysed cell’ Column), or Control matrices (i.e., without bacteria). The intact matrix was generated after late-log phase *Kp* Nevada was washed of extracellular beta-lactamase. Lysed samples were generated through sonication of *Kp* Nevada washed of extracellular beta-lactamase. Control samples were matrix obtained from the final wash procedure to confirm complete removal of extracellular beta-lactamase and to quantify thermodynamic degradation. (**b**) *Kp* Nevada total population bacterial counts (black dots and lines) and antibiotic-resistant subpopulations (colored dots and lines) quantified during the HFIM at an average starting concentration of 7.5 log_10_ CFU/ml mL. Scanning electron microscopy images of representative *Kp* Nevada from the indicated regimen and time point are shown in the inset.

Morphological alterations in Gram-negatives have been previously reported as a common response to antibiotics or environmental stress^29^. When *Kp* Nevada was grown in the presence of aztreonam monotherapy, we observed robust, antibiotic-induced filamentation that peaked at 6 h. As observed in time lapse microscopy (data not shown), elongation of rods in response to aztreonam exposure was seen at 2 h, and almost the entire population reverted to rod-shaped cells by 24 h. Reversion from filamentous to rod morphology occurred without the manual removal of drug pressure. Although *Kp* Nevada harbors four distinct β-lactamases that are enzymatically active against aztreonam, morphological changes indicative of penicillin binding proteins (PBPs) binding were nonetheless observed in response to drug exposure^12,30^. These findings suggested that at clinically relevant drug concentrations used in this study, sufficient PBP3 binding occurred to abrogate bacterial septation for at least the first 6 h of exposure. Thereafter, possibly, the metabolically active cell population nullifies the effect of antibiotic by effectively hydrolyzing aztreonam, resulting in the eventual reversion to rods. Impairment of cell wall synthesis by inhibiting PBPs is the primary mode of action for β-lactam antibiotics. In addition to producing antibiotic hydrolyzing enzymes, various gram-negative pathogens exhibit tolerance to β-lactam antibiotics by virtue of forming spheroplasts, i.e., cell wall deficient bacterial cells^25,31,32^. Rapid uptake and higher affinity of imipenem towards PBP2 lead to peak spheroplast formation by 2 h of antibiotic treatment (Fig. 1b)^33^. Reversion of spheroplasts to rods under static conditions can be explained by the increasing population of cells leading to higher enzymatic content resulting in enhanced degradation of imipenem. Fluorescence and scanning electron microscopy images from imipenem monotherapy showed a bacterial population comprised of mostly rods and few spheroplasts at 24 h (Fig. 1b). Time lapse microscopy revealed that, within 20 min of exposure, imipenem induced the formation of slightly rounded cells with bulging mostly from the center. By two hours, the effects of imipenem on spheroplast formation peaked, and most observable cells had converted into cell wall-deficient non-dividing spheroplasts. When *Kp* Nevada was exposed to the combination of aztreonam and imipenem under static conditions, blebbing was observed in the center of elongating filaments followed by a major burst in spheroplast formation within the first hour of drug exposure. Although aztreonam has been known to induce filamentation since its original discovery, the lack of filamentation observed under combination treatment with aztreonam and imipenem indicates the dominant effect of imipenem on morphology due to its rapid influx and inactivation of PBP1a/1b and PBP2 (Fig. 1b). Assessment of permeability using a previously established LC–MS/MS protocol showed aztreonam to have an estimated permeability coefficient of 83.1 nm/s for KP Nevada, imipenem displayed 207-fold higher permeability with mean permeability coefficient of 17,200 nm/s (Fig. 2a; see Supplemental for other parameter estimates).

To transiently withstand antibiotic pressure, many bacterial species are known to form phenotypic variants. However, little is known about the molecular determinants that accompany these morphotype changes^34^. β-Lactams are among the most trusted antibacterial agents and function by covalent binding and inactivation of PBPs, thereby disrupting the formation of intact cell wall/ peptidoglycan and facilitating cell death. Extensive studies on various PBP mutants in *E. coli* have shown that PBP2 and PBP3 with either PBP1a or 1b are essential for laboratory growth of regularly dividing rod-shaped cells^35^. Antibiotic tolerance and generation of viable-but-not-culturable cells have been reported in Gram-negatives treated with PBP2- and PBP3-targeted antibiotics. Imipenem has a relatively low binding affinity to PBP3, but it causes strong inhibition of the transpeptidase activity of PBP1a, PBP1b and PBP2^33^. Despite imipenem inactivating these PBPs, our RT PCR analyses revealed variable upregulation of *pbp1, pbp2, and pbp3* genes (Fig. 1c). We hypothesize that, as *Kp* Nevada harbors *bla*_NDM-1_ gene which produces the New Delhi metallo-β-lactamase (NDM-1) enzyme, it is likely that ongoing carbapenemase activity keeps periplasmic imipenem concentrations at sub-inhibitory concentration thereby inducing the SOS response leading to upregulation of these transcripts. Alternatively, the upregulation at transcript level could be to keep up with the translation rate as more and more PBPs are inactivated by the drug. Future studies will be aimed to measure the bound vs unbound PBPs in intact bacteria. Additionally, conforming to the phenotypic changes observed in the imipenem treated cells, there was a close to 3 times higher transcript level of *spy* (Spheroplast protein Y) as compared to the no drug control and a much more extensive (20-fold) increase in *spy* in cells that received combination therapy. Imaging studies showed that spheroplasts persisted for a longer period when exposed to aztreonam + imipenem therapy as compared to imipenem alone, possibly explaining the difference in *spy* transcript upregulation (Fig. 1d). SPY is known to be upregulated in spheroplasts and is hypothesized to be involved in the biogenesis of outer membrane proteins^36,37^. Inhibition of filament formation due to inactivation of PBP1a/1b and PBP2 by imipenem in the combination therapy could possibly be an imipenem mediated mechanism to inhibit persister formation and maximize killing.

Clinicians treating serious infections caused by XDR bacteria have successfully used ceftazidime/avibactam against carbapenem-resistant *Enterobacterales*; however, the combination lacks activity against strains producing NDM^38–40^. Although, combining aztreonam with ceftazidime/avibactam has demonstrated potent in vitro activity against Metallo-β-lactamase (MBL)-producing *Enterobacterales*, documented treatment failures and recurrent infections call for more effective options^41^. To fully define the early 3 log_10_ drop in viable counts as a result of aztreonam + imipenem in the time-kill assay, *Kp* Nevada was subsequently investigated in a 10-day hollow fiber infection model (HFIM) using cellulosic cartridges as described previously^14^. In addition to monotherapy controls, base therapy of aztreonam + ceftazidime/avibactam with and without imipenem was also tested to specifically dissect the contribution of imipenem. The following regimens were explored in the HFIM:

1. Growth Control
2. Aztreonam 2 g 2 h prolonged infusion (PI) every 8 h (Q8H)
3. Ceftazidime/ avibactam 2/0.5 g 2 h PI Q8H
4. Aztreonam 2 g 2 h PI Q8H + ceftazidime/ avibactam 2/0.5 g 2 h PI Q8H
5. Imipenem 3 g/day continuous infusion (CI)
6. Aztreonam 2 g 2 h PI Q8H + ceftazidime/ avibactam 2/0.5 g for 2 h PI Q8H + imipenem 3 g/day CI

At the average starting concentration of 7.5 log_10_ CFU/ml, monotherapy of aztreonam, imipenem or the ceftazidime/avibactam combination did not inhibit the growth of *Kp* Nevada, and the total counts closely mirrored the growth control throughout the course of the experiment. The extensive complement of chromosomal and plasmid-mediated β-lactamases (*bla*_NDM-1_, *bla*_CMY-6_, blaCTX_-M-15_, *bla*_SHV-28_) effectively hydrolyzed each β-lactam in monotherapy, which led to its active proliferation and monotherapy failure. However, the simultaneous administration of aztreonam with ceftazidime/avibactam produced an initial bactericidal activity of 3.3 log_10_ killing by 72 h, as compared to the starting inoculum, followed by regrowth to the system carrying capacity of approximately 10 log_10_ CFU/mL. By comparison, addition of imipenem to aztreonam + ceftazidime/avibactam resulted in an absolute reduction of 6.3 log_10_ CFU/mL by 72 h (Fig. 2b), a two fold higher extent of bacterial killing as compared to treatment without imipenem. After the first 24 h, combination regimens containing imipenem consistently exhibited increased extent and duration of bactericidal activity as compared to combinations without imipenem. Total counts from 30 h through 168 h vividly demonstrated that the presence of imipenem contributed synergistically to the prolonged antimicrobial action. After treatment withdrawal, the average bacterial concentration between 30 and 168 h was 3.2 log_10_ CFU/mL for aztreonam + ceftazidime/avibactam in combination with imipenem, as compared to 5.1 log_10_ CFU/mL for aztreonam + ceftazidime/avibactam (Fig. 2b). This is the first report where the addition of carbapenem to the base regimen of aztreonam plus ceftazidime/avibactam has addressed the effects of persistent cell populations, resulting in twofold higher extent of bacterial killing. In addition to improved bacterial killing, the addition of imipenem resulted in an enhanced post-antibiotic effect. During the reversion phase of the experiment starting at 168 h, aztreonam + ceftazidime/avibactam experienced bacterial regrowth from 5.9 log_10_ CFU/mL to 9.5 log_10_ CFU/mL within 24 h of treatment withdrawal. However, this rate of reversal was not observed for the imipenem-containing combination for 48 h after treatment withdrawal. These data suggest that the continuous infusion of imipenem to the trio base regimen compounds bacterial killing, while simultaneously extending the post-antibiotic effect period. We hypothesize that the extended post-antibiotic effect could be attributed to decreased persister formation and slower recovery of the bacterial cell wall. By generating fewer viable but non-culturable persisters and extending the time needed for the few remaining persisters to revert, there is a significantly extended post-antibiotic effect period. Ultimately, these data support that upon addition of imipenem to aztreonam + ceftazidime/avibactam, formation of cell wall devoid spheres could possibly be a mechanism to prevent persister filament formation that improves the clearance of PDR *K. pneumoniae* CDC Nevada*.* Real-time heteroresistance to polymyxin B, aztreonam/avibactam, and ceftazidime was assessed via population analysis profiles (PAPs). PAPs showed that in all cases the resistant subpopulations capable of growing on agar containing ceftazidime (2 mg/L, 8 mg/L, 32 mg/L) or polymyxin B (0.5 mg/L, 2 mg/L) were largely represented by the total counts. There was little heterogeneity in bacterial resistance with either aztreonam or lower concentration of polymyxin B. The aztreonam/avibactam PAPs showed complete suppression compared with the growth control, indicating that there was no proliferation of resistance due to the treatment, as bactericidal activity was observed throughout, even in the least successful regimen of ceftazidime/avibactam 2/0.5 g 2 h PI Q8H and aztreonam 2 g 2 h PI Q8H (Supplemental).

## Future directions

Non-replicating persister formation is a survival strategy by bacteria to evade total eradication, while waiting for more favorable environmental conditions for growth and replication^42^. In our microscopy findings, we observed drug induced persister formation that had an elongated lag-phase before reverting to a replicating, rod-shaped morphology upon drug removal. Although the otherwise recalcitrant spheroplasts build resistance in the case of monotherapy, we hypothesize that the absence of cell wall in spheroplasts ultimately make these persistent and tolerant bacteria vulnerable to damage by other cell-wall or cell-membrane active drugs, which stress the cell barrier beyond the bacteria’s ability to compensate. Future hollow fiber infection model studies will focus on a more targeted approaches to eliminating persisters utilizing these foundational data. Regimes that can target cell wall deficient spheroplasts will possibly achieve better clearance without relapse. Additionally, since a large subset of persister cells exhibit altered metabolism, future regimens would aim at clearing subpopulations with altered phenotypes that interfere with critical metabolic processes that are maintained even under persistence, including protein synthesis, protein degradation, and direct DNA damage^43^. Overall, these studies have set the cornerstone for therapeutic options that dually target antibiotic persistence and resistance as a new paradigm to combat Gram-negative bacteria that are resistant to all antibiotics.

### Supplementary Information

Supplementary Information.

## Data Availability

*Kp* investigated in this study has been previously characterized by de Man et al.^12^. Findings from the genomic characterization of this isolate were placed under BioProject PRJNA391323 (https://www.ncbi.nlm.nih.gov/bioproject/PRJNA391323). The chromosome, IncA/C2, IncFIB(pKPHS1), and IncFIB(K) genome sequences can be found under GenBank accession numbers CP022127, CP022126, CP022128, and CP022125, respectively^12^.
