# Supplementary material for: Next generation antibiotic combinations to combat pan-drug resistant Klebsiella pneumoniae
Source: Sci Rep. 2024 Feb 7;14:3148. doi: 10.1038/s41598-024-53130-z (PMC10850076; doi:10.1038/s41598-024-53130-z)
Supplement: Supplementary file 1 — Supplementary Information. [file 41598_2024_53130_MOESM1_ESM.docx]

**SUPPLEMENTAL**

**Table S1:** Additional parameter estimates for permeability.

| **Drug** | **P (%RSE)** | **CL_int_^a^ (%RSE)** | **K_m_ (%RSE)** | **T_deg_^b^ (%RSE)** | **Residual Variance^c^**  **(%RSE)** |
| --- | --- | --- | --- | --- | --- |
|  | **nm/s** | **mL/min** | **μg/mL** | **hour** |  |
| **Aztreonam** | 4.69 (10.3%) | 10.3 (11.4%) | 13.1 (8.3%) | 15,400 (15.7%) | SD_s_ = 0.0526 (15.5%)  SD_i_ = 0.157 (19.3%) |
| **Piperacillin** | 124 (11.8%) | 15.6 (10.4%) | 5.26 (20.1%) | 4730 (10.0%) | SD_s_ = 0.241 (15.5%) |
| **Ceftazidime** | 152 (5.29%) | 1.39 (8.80%) | 20.7 (14.3%) | 4680 (6.00%) | SD_s_ = 0.187 (11.1%) |
| **Cefepime** | 161 (21.3%) | 0.564 (24.2%) | 9.99 (12.1%) | 2480 (8.50%) | SD_s_ = 0.264 (8.4%) |
| **Imipenem** | 22,700 (3.14%) | 9.11 (8.80%) | 17.8 (22.4%) | 420 (9.50%) | SD_s_ = 0.338 (14.2%) |
| **Meropenem** | 2010 (6.49%) | 13.9 (12%) | 9.11 (16.4%) | 1810 (7.00%) | SD_s_ = 0.0527 (22.9%)  SD_i_ = 0.134 (27.5%) |
| ^a^CL_int_ = V_max_ / K_m_  ^b^k_deg_ = ln(2) / T_deg_  ^c^Var(Y) = (SD_s_ • Y + SD_i_)^2^  **Table S2:** Primers used for RT-PCR   \| **Transcript** \| **Forward Primer** \| **Reverse Primer** \| \| --- \| --- \| --- \| \| *pbp1* \| TGATTGCCGAAGATGGCACT \| ATTGTTGGTGGTCCCGGTTT \| \| *pbp2* \| AGATCCGGTGCGCGTTATAG \| ATCGCCAAAGACGGCATGTA \| \| *pbp3* \| TACAGAGTCATCCCCACGCT \| TTTCTCCACCCCTTCGATGC \| \| *spy* \| GTTGCCTCTACTCTGGCTCTG \| GAGCATCGGTCAGGTTCAGG \| \| *proC* \| TATCGCCCAGCACTTTGGTC \| CAGGTACAGCCGGGACAAAT \| \| *gyrA* \| GTGACCCGTCGTACGATTTT \| GATAATCGGGTCGATGTTGG \| | | | | | |

**Figure S1:** Population analysis profiles for resistant subpopulations represent growth on MHA plates imbued with polymyxin B or aztreonam/avibactam or ceftazidime throughout the HFIM experiment.

To assess permeability, serial samples were collected to quantify degradation rates of each beta-lactam at 9 different time points: 1, 2, 5, 10, 15, 30, 60, 90, and 120 mins. Samples were diluted 1:5 in methanol+IS stock to precipitate proteins then centrifuged at 15000 rcf for 5 mins. Finally, supernatant from each sample was diluted 1:10 in HPLC water for subsequent chromatographic separation. Separation was performed on Shimadzu Prominence HPLC then quantified on a Sciex API 3000 Triple Quadrupole mass spectrometer with ESI ionization positive mode. Drugs were separated on a Waters XSelect C18 3.5um 2.1x100mm column. The mobile phase consisted of 10mM ammonium acetate pH 5 (Phase A) and 90/10 acetonitrile/water + 10mM ammonium acetate (Phase B). Gradient elution was used and was based on compounds that were used. For aztreonam, ceftazidime, and cefepime, 2% B from 0 to 4.9 mins, 80%B 5 to 6 mins, 95%B 6.1 to 10 mins, and 2%B 10.1 to 14 mins. For meropenem, imipenem, and piperacillin, 1% B from 0 to 5.9 mins, 80%B 6 to 6.1 mins, 95%B 6.1 to 10 mins, and 2%B 10.1 to 14 mins.

Multiple reaction monitoring was used to quantify each beta-lactam and optimized to identify the following parent/product ions: aztreonam m/z 436 to 312; ceftazidime m/z 547 to 468, cefepime m/z 481 to 395; imipenem m/z 300 to 169; meropenem m/z 284 to 140; and piperacillin m/z 518 to 142. Isotopically labeled internal standards were included for each beta-lactam, except for imipenem which was normalized using isotopically labeled ceftazidime-d6. (all from Toronto Research Chemicals, Toronto, CA) The lower limit of quantification was determined to be the following for each drug: 0.1 mg/L aztreonam, 0.1 mg/L ceftazidime, 0.1 mg/L cefepime, 0.1 mg/L imipenem, 0.1 mg/L meropenem, and 0.3 mg/L piperacillin.
